# Key hepatic signatures of human and mouse nonalcoholic steatohepatitis: A transcriptome–proteome data meta-analysis

**DOI:** 10.3389/fendo.2022.934847

**Published:** 2022-10-04

**Authors:** Jeong Joo Pyo, Yongsoo Choi

**Affiliations:** ^1^ Natural Product Research Center, Korea Institute of Science and Technology (KIST), Gangneung, South Korea; ^2^ Division of Bio-Medical Science & Technology, KIST School, Korea University of Science and Technology, Seoul, South Korea

**Keywords:** nonalcoholic fatty liver disease, nonalcoholic steatohepatitis, transcriptomics, proteomics, cross-species analysis

## Abstract

**Background:**

Despite the global prevalence of nonalcoholic fatty liver disease (NAFLD), its pathophysiology remains unclear. In this study, we established highly confident nonalcoholic steatohepatitis (NASH) gene signatures and evaluated the pathological mechanisms underlying NASH through a systematic meta-analysis of transcriptome and proteome datasets obtained from NASH patients and mouse models.

**Methods:**

We analyzed NASH transcriptome datasets from 539 patients and 99 mice. A whole-liver tissue proteome dataset was used to confirm the protein level dysregulation of NASH signatures significant in both humans and mice.

**Results:**

In total, 254 human and 1,917 mouse NASH gene signatures were established. Up-regulated genes of 254 human signatures were associated with inflammation, steatosis, apoptosis, and extracellular matrix organization, whereas down-regulated genes were associated with response to metal ions and lipid and amino acid metabolism. When different mouse models were compared against humans, models with high fat and high fructose diet most closely resembled the genetic features of human NAFLD. Cross-species analysis revealed 66 genes that were concordantly dysregulated between human and mouse NASH. Among these, 14 genes were further validated to be dysregulated at the protein level. The resulting 14 genes included some of the well-established NASH associated genes and a promising NASH drug target. Functional enrichment analysis revealed that dysregulation of amino acid metabolism was the most significant hepatic perturbation in both human and mouse NASH.

**Conclusions:**

We established the most comprehensive hepatic gene signatures for NASH in humans and mice to date. To the best of our knowledge, this is the first study to collectively analyze the common signatures between human and mouse NASH on a transcriptome–proteome scale.

## Introduction

Nonalcoholic fatty liver disease (NAFLD), defined by the presence of abnormal liver fat (steatosis in ≥ 5% of hepatocytes) in the absence of secondary causes of fatty liver, is the most common form of chronic liver disease worldwide (1). NAFLD encompasses a spectrum of conditions, from the simple nonalcoholic fatty liver (NAFL) to the more severe nonalcoholic steatohepatitis (NASH), which is hallmarked by inflammation and hepatocyte ballooning (1). The global prevalence of NAFLD is rapidly increasing, concurrent to the global epidemics of obesity and type 2 diabetes mellitus (T2DM) (2). It is estimated that 25% and 5% of the general population have NAFLD and NASH, respectively (2). Due to the high propensity of NASH to further develop into cirrhosis and hepatocellular carcinoma (HCC), NASH is becoming the leading cause of liver transplantation, but approved therapies have not yet been developed (3).

The pathogenesis of NASH is a complex multi-etiological process associated with genetic, epigenetic, metabolic, and environmental factors (4). Two gene variants, patatin-like phospholipase domain-containing protein 3 (*PNPLA3*) and transmembrane 6 superfamily member 2 (*TM6SF2*), have been validated to be strongly associated with NASH (4). Obesity and insulin resistance are key pathogenic factors in NAFLD, and T2DM is a well-established risk factor for the rapid progression of NAFL to NASH, cirrhosis, or HCC (5). Recent studies revealed an inverse correlation between diet quality and NAFLD prevalence (6), and regular consumption of fructose promoted hepatic *de novo* lipogenesis in a double-blind, randomized clinical trial (7). Despite many efforts to understand the disease, the complete pathophysiology of NASH remains unclear. Current options for managing NAFLD include bariatric surgery or lifestyle modification, such as exercise or diet control (1). These methods have proven to be effective in resolving NASH or even mild fibrosis, but are only applicable to a limited number of patients (1). Thus, additional studies are urgently needed to understand the pathogenesis of NASH for successful identification of therapeutic NASH targets and the development of corresponding drugs.

Animal models, particularly those used to study human diseases, offer valuable opportunities to researchers to experimentally investigate the pathophysiology of diseases *via* genetic or dietary interventions. NASH is regarded as a metabolic syndrome, and various mouse diet models have been developed to mimic human NASH. Although mouse models cannot represent the full spectrum of human NASH due to the genetic differences between the species, consistent dysregulation patterns of orthologous genes or proteins across the species could offer valuable insights into the disease pathogenesis. High-throughput technologies, such as transcriptomics and proteomics, are invaluable tools for providing a holistic view of biological systems. Transcriptomics, with its high-resolution and genome-wide capacity, has enabled researchers to query global gene expression patterns and infer protein abundances from mRNA abundances. Liquid chromatography-tandem mass spectrometry-based proteomics can be used to directly measure the abundance of proteins and identify post-translational modifications. Combining these two approaches can better depict biological dysregulations within cells or tissues (8). Although several previous studies have individually reported transcriptomic or proteomic changes in NASH, none of them have collectively analyzed the common signatures between human and mouse NASH on a transcriptome–proteome scale.

Therefore, in this study, we established comprehensive gene signatures of human and mouse NASH and profiled significantly aberrant genes to better understand the pathological mechanisms underlying NASH. To this end, we performed a systematic meta-analysis of publicly available transcriptome and proteome datasets from liver tissues of patients with NASH and mouse models.

## Methods

This meta-analysis was performed according to the PRISMA (Preferred Reporting Items for Systematic Reviews and Meta-Analyses) guidelines (9). A detailed checklist is provided in the **Supplementary Materials**.

### Data sources and search strategies

A systematic dataset search was conducted up to March 2021. To obtain transcriptome study datasets, publicly available databases ArrayExpress (https://www.ebi.ac.uk/arrayexpress) and Gene Expression Omnibus (GEO) (https://www.ncbi.nlm.nih.gov/geo) were utilized. The following keywords were used to search human NASH transcriptome datasets: “Homo sapiens,” “RNA assay,” “Expression profiling,” “Nonalcoholic fatty liver disease,” “Nonalcoholic steatohepatitis,” “NAFLD,” and “NASH.” After removing duplicates, 9 datasets from ArrayExpress and 78 datasets from GEO were identified. Two additional datasets were identified through a reference search. When searching mouse NASH model datasets, the following keywords were used: “Mus musculus,” “RNA assay,” “Expression profiling,” “Nonalcoholic fatty liver disease,” “Nonalcoholic steatohepatitis,” “NAFLD,” and “NASH.” After removing duplicates, 7 datasets from ArrayExpress and 187 datasets from GEO were identified.

Proteome study datasets were searched using the publicly available ProteomeXchange database (http://www.proteomexchange.org). The keywords “NAFLD” or “NASH” were first used to search the datasets, and the results were subsequently filtered according to species. After removing duplicates, 8 human datasets and 10 mouse model datasets were identified.

### Dataset selection process and eligibility criteria

**Figure 1:**
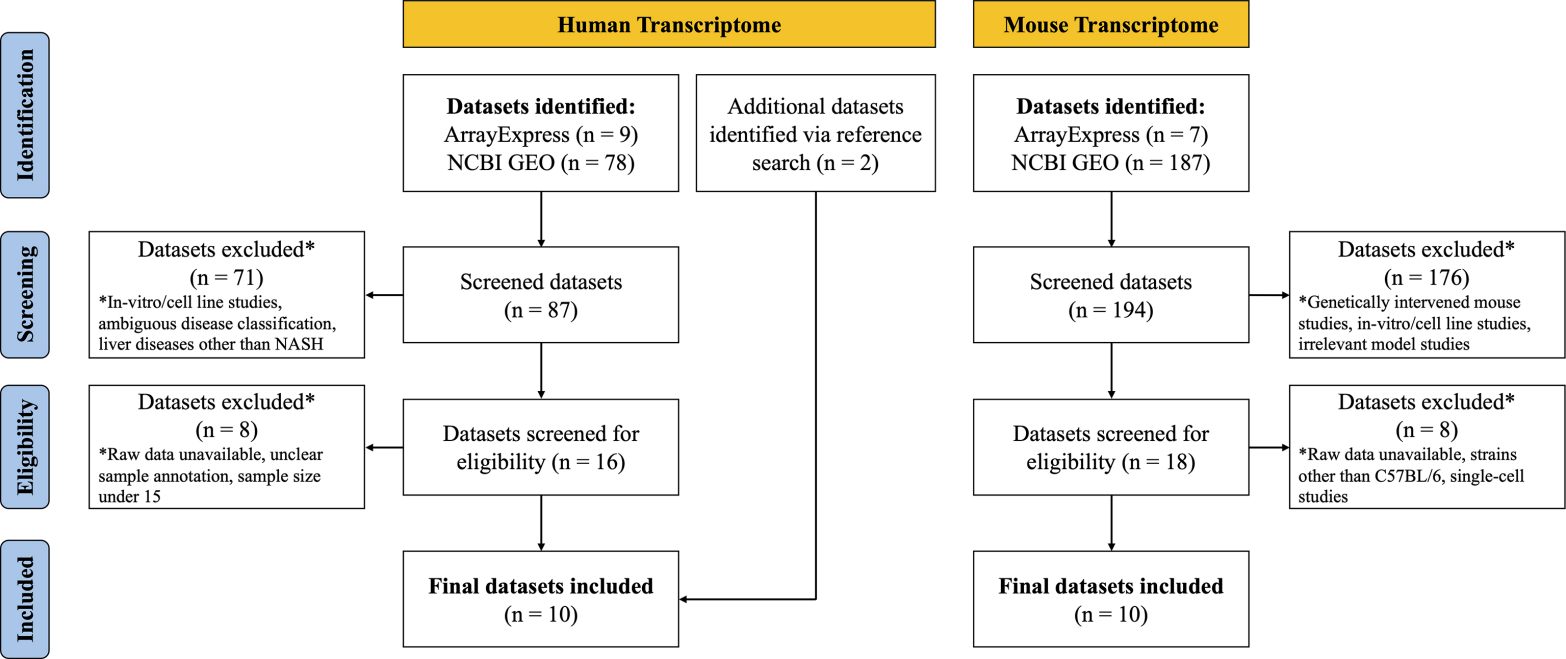
Flow diagram on the selection process of human and mouse NASH transcriptome datasets.

After systematic dataset identification, the datasets were further screened and excluded according to the following criteria. For human NASH studies, datasets were excluded when they corresponded to: (a) *in vitro*/cell line studies; (b) ambiguous disease classification; (c) studies involving other liver diseases (such as hepatitis, cirrhosis or HCC); (d) unavailability of raw data; (e) unclear sample annotation; and (f) total sample size ≤ 15. For the NASH mouse model studies, datasets were excluded when they corresponded to: (a) genetically intervened mouse studies; (b) *in vitro*/cell line studies; (c) NASH irrelevant model studies; (e) unavailability of raw data; (f) studies with mouse strains other than C57BL/6; and (g) single-cell transcriptome studies (**Figure 1**).

For human NASH proteome studies, datasets were excluded when they corresponded to: (a) *in vitro*/cell line studies; (b) studies with samples other than liver tissue (e.g., plasma); and (c) enriched peptide studies. NASH mouse model proteome study datasets were excluded when they corresponded to: (a) genetically intervened mouse studies; (b) *in vitro*/cell line studies; (c) studies with samples other than liver tissue; and (d) enriched peptide studies (**Supplementary Figure 1**).

### Transcriptome dataset processing

Nine human (GSE33814 (10), GSE37031 (11), GSE48452 (12), GSE49541 (13), GSE61260 (14), GSE63067 (15), GSE66676 (16), GSE126848 (17), E-MEXP-3291 (18)) and 10 murine (GSE35961 (19), GSE43106 (20), GSE52748 (21), GSE93819 (22), GSE94593 (23), GSE119340 (24), GSE120977 (25), GSE137449 (26), GSE145665 (27), GSE148849 (28)) transcriptome datasets were subjected to further analysis. The raw data or processed data from each study were downloaded from the respective data repositories. The analysis platform for each dataset was heterogeneous and consisted of microarray and RNA sequencing data from multiple vendors. Due to the variability of platforms and discrepancies in sample handling or disease assessment of each study cohort, each dataset was analyzed separately. Affymetrix microarray raw data (.CEL files) were downloaded, and gene expression values were processed using Affymetrix Expression Console software (Affymetrix, Santa Clara, CA, USA) with robust multiarray averaging (RMA) or microarray analysis suite 5.0 (MAS5) normalization. Processed Illumina BeadChip data were downloaded from NCBI GEO and probeset intensity normalization was performed using the ACTB and GAPDH genes as internal controls. In most cases, RNA sequencing read counts raw data were provided by NCBI GEO or the authors who published the studies. Normalization of the read counts and differentially expressed gene (DEG) selection were performed as downstream analyses if necessary. If raw counts were not available, RAW data were retrieved from Sequence Read Archive and further processed using the Galaxy platform. Detailed information on the acquisition, processing, normalization, and analysis method for each dataset is shown in **Supplementary Table 1**.

DEGs from each transcriptome dataset were individually selected as described by Pyo et al. (29). Briefly, after normalization of each gene expression value, the fold change and Benjamini–Hochberg adjusted *p*-value were calculated between the normal and NASH groups. Up-regulated DEGs were determined if the fold change (NASH/Normal) was greater than 1.7 and *p*-value was lower than 0.05. Down-regulated DEGs were determined if the fold change was lower than 0.7 and *p*-value was lower than 0.05. In the case of GSE49541, the normal group cohort was not available; hence, the fold change and *p*-value were determined by comparing the advanced NAFLD group with the mild NAFLD group. Although raw data were not available for the study published by Dali-Youcef et al. (30), a complete list of up-regulated and down-regulated DEGs was available in the manuscript. The DEGs were extracted and used for further analysis, affording a total of 10 human NASH transcriptome datasets that were used in this study.

### Proteome dataset processing

Raw data (.RAW files) of the NASH mouse model liver proteome study PXD013423 (31) were downloaded from ProteomeXchange. Proteome Discoverer 2.3 (Thermo Fisher Scientific, Hanover Park, IL, USA) was used for the precursor quantification and label-free quantitative analyses. The UniProt mouse proteome reference (https://www.uniprot.org, March 2021 version) was used for MS2 peptide spectral matching. The assignment of MS2 spectra was carried out using the SEQUEST algorithm, and peptide hits were filtered at a maximum of 1% FDR using the Percolator algorithm. Carbamidomethylation of cysteine was set as static modification, whereas methionine oxidation and N-terminal acetylation were set as dynamic modifications. Full trypsin specificity with up to two missed cleavage sites was applied. Mass tolerance for precursor and fragment ions were set at 10 ppm and 0.02 Da, respectively.

Differentially expressed proteins (DEPs) were determined using the SEQUEST search parameters. Valid up-regulated DEPs were determined when SEQUEST HT score ≥ 30, protein coverage ≥ 20, and protein abundance of NASH/control ≥ 1.7. Down-regulated DEPs were determined when SEQUEST HT score ≥ 30, protein coverage ≥ 20, and protein abundance of NASH/control ≤ 0.7.

### Establishing key common human and mouse hepatic NASH signatures

First, the selected up-regulated and down-regulated DEGs from the 10 human NASH datasets were merged based on the gene symbols. In this process, DEGs showing different dysregulation pattern between studies were excluded. Then, the frequency score (how many times a certain gene was listed as a DEG among the 10 datasets) of each DEG was calculated. Only the DEGs with frequency ≥ 3 constituted the human NASH signature (described in Section 2.6). Next, to compare the human NASH signature with that of mice, dysregulated DEGs from 10 NASH mouse model datasets were merged as described for the human datasets, and the frequency of each DEG was calculated. DEGs with a frequency ≥ 4 comprised the mouse NASH signature. Finally, NASH signatures of both humans and mice were integrated using BioMart orthologous gene annotation. Genes showing a consistent dysregulation pattern between the two species constituted a common hepatic NASH signature. To validate the NASH signatures at the protein level, the UniProt accession numbers of selected DEPs from PXD013423 were converted into gene symbols, and both profiles were combined. The final common NASH signature comprised the DEG frequency from human and mouse transcriptome datasets and fold change values from a mouse proteome dataset (**Supplementary Data**).

### Degree of confidence of DEGs in multiple transcriptome dataset meta-analysis

The selection of DEGs from individual transcriptome datasets involved a fold change cutoff and statistical significance validation. Nevertheless, DEG lists from different cohorts of the same disease showed significant discrepancies. This may be due to heterogeneous patient groups (e.g., ethnicity), variability in disease diagnosis, assay platform differences, technical bias, instrumental error, or inherent limitations in statistical assumptions. Such problems can be partly overcome by identifying consistent gene dysregulation patterns across multiple independent cohort studies. Furthermore, when analyzing multiple datasets, the DEG frequency score can be added as statistical dimension to evaluate the confidence of the DEGs.

The previous transcriptome datasets of humans and mice were used to evaluate the degree of confidence of the DEGs. However, rather than selecting DEGs based on their fold changes and *p*-values in individual datasets, genes were randomly selected (number of DEGs and randomly selected genes (RSGs) was matched for each dataset). Subsequently, RSGs were merged based on the gene symbols, and the frequency score of each gene was calculated. This process was bootstrapped 1,000 times, and the results were recorded each time. Finally, the median value of 1,000 times bootstrap resampling was calculated. (**Supplementary Figure 2**). The degree of confidence of DEGs per frequency score was calculated using the following formula:


$$Degree\;of\;Confidence=100-\frac{\mathrm{Number}\;of\;RSGs/\mathrm{Number}\;of\;total\;RSGs}{\mathrm{Number}\;of\;DEGs/\mathrm{Number}\;of\;total\;DEGs}\times 100$$

DEGs with a degree of confidence ≤ 50 were classified as “Not significant,” confidence between 100 and 50 were classified as “Fair confident,” and confidence of 100 were classified as “High confident” DEGs.

### Gene Ontology biological process enrichment analyses

Gene Ontology (GO) biological process (BP) enrichment analysis was performed as described by Pyo et al. (28). Enriched GO BP for corresponding DEGs was determined by comparing the frequency of genes annotated by GO BP terms in a group of DEGs with those in the entire set of genes in the human reference list. The GO annotation files were downloaded from the Gene Ontology Consortium webpage (http://www.geneontology.org), and the March 2021 version of the GO BP terms was used for the analysis. A 2 × 2 contingency table was constructed to compare the frequency of DEGs annotated by the GO BP terms, with the number of genes annotated by these terms in the total 20,595 human gene reference. The 2 × 2 contingency table was analyzed for the calculation of *p*-values using the X^2 test (frequency ≥ 5) or Fisher’s exact test (frequency< 5).

### Software and statistical analyses

Statistical analyses were performed using the GraphPad Prism 8.0 software (GraphPad Software Inc., San Diego, CA, USA). Filtration, classification, and integration of transcriptome and proteome datasets were conducted using R software (version 4.0.3), specifically the packages included in “tidyverse”. The transcriptome profile datasets were visualized in a heatmap using the heatmap.2 function in “gplots” package, and the Venn diagram was produced using the “VennDiagram” package. The heatmap and volcano plot for the proteome dataset were drawn using Proteome Discoverer 2.3.

## Results

### Description of included datasets

Details on the selection process of human and mouse NASH transcriptome datasets can be found in the flow diagram (**Figure 1**). A total of 10 human (539 patients) and 10 mouse model (99 mice) studies were included in this study. All the 10 human NASH datasets have been published and represent patients of multiple ethnicities. All included participants underwent liver biopsy, and NAFLD was diagnosed histologically (**Table 1**). The 10 NASH mouse model datasets were also previously published, used the wild-type C57BL/6 strain, and comprised multiple dietary intervention methods including a methionine- and choline-deficient diet with high-fat diet (MCDHFD), high-fat diet (HFD), NASH-inducing diet (ND), high-cholesterol high-cholate high-fat diet (CLD), high-fat high-sugar diet (HFHSD), fat fructose cholesterol diet (FFCD), choline-deficient L-amino acid-defined high-fat diet (CDAHFD), and fast food diet (FFD) with varying degrees of duration. Details of the diet composition and experimental design are summarized in **Table 2**.

**Table 1:** Study characteristics of human NASH transcriptome datasets used in this study.

Study	Country	Patient group	Disease assessment	Platform	Data availability
**Starmann et al., 2012 (** 10)	Germany	Normal 13 Steatosis 19 NASH 12	Biopsy	Illumina Human WG-6 v3.0expression beadchip	GEO GSE33814
**López-Vicario et al., 2014 (** 11)	Spain	Normal 7 NASH 8	Biopsy	Affymetrix Human GenomeU133 Plus 2.0 Array	GEO GSE37031
**Ahrens et al., 2013 (** 12)	Germany	Normal 14 Obese 27 Steatosis 14 NASH 18	Biopsy	Affymetrix Human Gene 1.1 ST Array	GEO GSE48452
**Moylan et al., 2014 (** 13)	USA	Mild NAFLD 40 Advanced NAFLD 33	Biopsy	Affymetrix Human Genome U133 Plus 2.0 Array	GEO GSE49541
**Horvath et al., 2014 (** 14)	USA	Normal 38 Obese 24 NAFLD 23 NASH 24	Biopsy	Affymetrix Human Gene 1.1 ST Array	GEO GSE61260
**Frades et al., 2015 (** 15)	Sweden	Normal 7 Steatosis 2 NASH 9	Biopsy	Affymetrix Human Genome U133 Plus 2.0 Array	GEO GSE63067
**Xanthakos et al., 2015 (** 16)	USA	Normal 34 Steatosis 26 Borderline NASH 5 NASH 2	Biopsy	Affymetrix Human Gene 1.0 ST Array	GEO GSE66676
**Suppli et al., 2019 (** 17)	Denmark	Normal 14 Obese 12 NAFLD 15 NASH 16	US, Biopsy	Illumina NextSeq 500	GEO GSE126848
**Lake et al., 2015 (** 18)	USA	Normal 19 Steatosis 10 NASH 16	Biopsy	Affymetrix Human Gene 1.0 ST Array	ArrayExpress E-MEXP-3291
**Dali-Youcef et al., 2019 (** 30)	France	Normal 10 Obese 10 Steatosis 10 NASH 8	Biopsy	Agilent Human GE 8x60K	N/A

NASH, nonalcoholic steatohepatitis; NAFLD, nonalcoholic fatty liver disease; US, ultrasonography; N/A, not available.

**Table 2:** Study characteristics of mouse NASH model transcriptome datasets used in this study.

Study	Diet model	Diet composition	Group	Platform	Data availability
**Kita et al., 2012 (** 19)	MCDHFD, 8 weeks	Methionine- and choline- deficient diet with 60% fat	Normal 4 NASH 4	Affymetrix Mouse Genome 430 2.0 Array	GEO GSE35961
**Kahle et al., 2013 (** 20)	HFD, 3 weeks	15% calories from casein, 27% calories from starch, maltose dextrin, cellulose 58% calories from soybean oil, safflower oil	Normal 8 NASH 7	Affymetrix Mouse Gene 1.0 ST Array	GEO GSE43106
**Dorn et al., 2014 (** 21)	ND, 12 weeks	15% pork lard, 15% beef tallow, 4% palmitic acid, 4% stearic acid, 0.2% cholesterol, 30% sucrose	Normal 4 NASH 4	Affymetrix Mouse Gene 1.1 ST Array	GEO GSE52748
**Kobori et al., 2017 (** 22)	CLD, 12 weeks	60% calories from fat, 1.25% cholesterol, 0.5% sodium cholate	Normal 5 NASH 5	Affymetrix Mouse Genome 430 2.0 Array	GEO GSE93819
**Maradana et al., 2018 (** 23)	HFHSD, 14 weeks	N/A	Normal 3 NASH 3	Illumina HiSeq 4000	GEO GSE94593
**Xiong et al., 2019 (** 24)	FFCD, 25 weeks	40% fat, 22% fructose, 2% cholesterol	Normal 3 NASH 3	Illumina HiSeq 2500	GEO GSE119340
**Min-DeBartolo et al., 2019 (** 25)	CDAHFD, 12 weeks	L-amino acid diet with 45 kcal%fat with 0.1% methionine and no added choline	Normal 5 NASH 5	Illumina HiSeq 4000	GEO GSE120977
**Heintz et al., 2020 (** 26)	CDAHFD, 8 weeks	18% protein, 62% fat, 20% carbohydrates, 0.1% methionine	Normal 4 NASH 4	Illumina NovaSeq 6000	GEO GSE137449
**Lu et al., 2020 (** 27)	HFD, 24 weeks	60% fat, 20% carbohydrate, 20% protein	Normal 5 NASH 5	Illumina HiSeq 4000	GEO GSE145665
**Bates et al., 2020 (** 28)	FFD, 21 weeks	17% kcal protein, 40% kcal fat, 43% kcal carbohydrate	Normal 8 NASH 10	Illumina HiSeq 2500	GEO GSE148849

NASH, non-alcoholic steatohepatitis; MCDHFD, Methionine- and choline-deficient diet with high-fat diet; HFD, high-fat diet; ND, NASH-inducing diet; CLD, high-cholesterol high-cholate high-fat diet; HFHSD, high-fat high-sugar diet; FFCD, fat fructose cholesterol diet; CDAHFD, choline-deficient L-amino acid defined high fat diet; FFD, fast food diet; N/A, not available.

### Hepatic gene signature of human NASH

**Figure 2:**
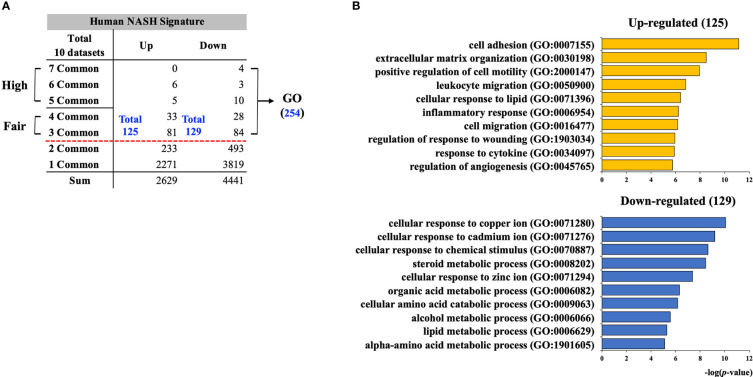
Establishment of human NASH signature and functional enrichment analysis. **(A)** Individually selected DEGs from 10 human NASH datasets were merged based on the gene symbols and the frequency score of each DEG was calculated. Total 254 genes (125 up-regulated and 129 down-regulated) were consistently dysregulated in at least three datasets. DEGs with frequency scores of 3 and 4 were classified as “Fair confident”, and frequency scores of 5 to 7 were classified as “High confident” genes. **(B)** GO BP enrichment analysis result for 254 human NASH signatures. NASH, nonalcoholic steatohepatitis; GO BP, gene ontology biological process.

First, to establish the hepatic gene signature of human NASH, the selected up-regulated and down-regulated DEGs from 10 individual NASH studies were merged based on the gene symbols. Next, 535 DEGs showing heterogeneous dysregulation patterns between the studies were excluded. Consequently, a total of 7,070 (2,629 up-regulated and 4,441 down-regulated) DEGs was compiled. The frequency score for each DEG was calculated. One or two overlapping DEGs were regarded as statistically insignificant, and only DEGs with a frequency ≥ 3 were considered as valid, resulting in 254 human NASH gene signatures (**Figure 2A**). These genes were further divided into “Fair confident” and “High confident” gene groups according to the degree of confidence per each frequency score. (**Supplementary Figure 2A**). Accordingly, 28 genes were identified as dysregulated in human NASH with high confidence (**Supplementary Table 2**).

### Functional enrichment analysis on 254 human NASH signatures

To gain a holistic view of the biological processes associated with the 254 human NASH signatures, GO BP enrichment analysis was performed. As a result, 125 up-regulated genes were significantly associated with inflammation (GO:0007155; 0050900; 0006954; 0034097), steatosis (GO:0071396), apoptosis (GO:1903034), and extracellular matrix (ECM) organization (GO:0030198) (**Figure 2B** and **Supplementary Table 3**). Not surprisingly, hepatic steatosis, inflammation, and apoptosis are the three main histological hallmarks of NASH, and the upregulation of ECM synthesis is a sign of NASH with fibrosis. Thus, we concluded that the up-regulated signature of human NASH fairly represents the overall histological features of NASH. In the analysis of the 129 down-regulated signatures, biological processes related to cellular response to metal ions (GO:0071280; 0071276; 0071294), lipid metabolism (GO:0008202; 0006629), and amino acid metabolism (GO:0009063; 1901605) were significantly enriched (**Figure 2B** and **Supplementary Table 4**).

### Comparative analysis of gene expression profiles of different NASH mouse models

**Figure 3:**
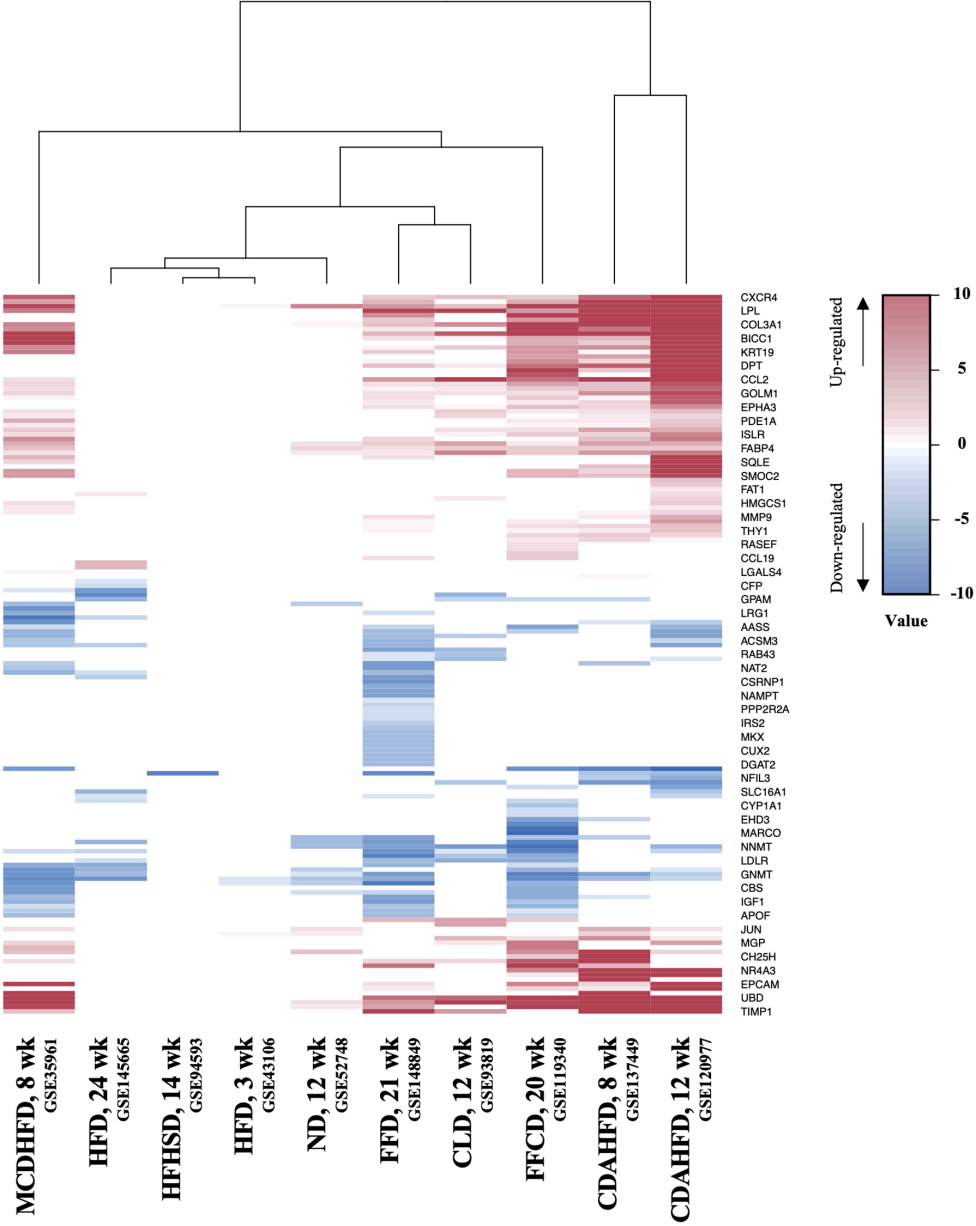
Heatmap of gene expression profiles of different NASH mouse models. DEGs from 10 mouse NASH datasets were merged based on the gene symbols, and the fold change values of dysregulated genes were normalized. Gradient of red color represents high expression of genes, whereas gradient of blue color represents low expression of genes in mouse NASH group. MCDHFD, methionine- and choline-deficient diet with high-fat diet; HFD, high-fat diet; HFHSD, high-fat high-sugar diet; ND, NASH-inducing diet; FFD, fast food diet; CLD, high-cholesterol high-cholate high-fat diet; FFCD, fat fructose cholesterol diet; CDAHFD, choline-deficient L-amino acid defined high fat diet.

Next, to establish the hepatic gene signature of mouse NASH, up-regulated and down-regulated DEGs from 10 mouse model studies were merged, and the frequency of each DEG was calculated. For the mouse datasets, DEGs with a frequency ≥ 4 were statistically significant, resulting in 1,917 mouse NASH gene signatures (**Supplementary Figure 3**). These genes were divided into “Fair confident” and “High confident” genes as well (**Supplementary Figure 2B**). Since NASH model studies consisted of multiple dietary intervention methods of varying durations, we first compared the gene expression profiles of different dietary model studies. As expected, we observed considerable disparities between different NASH models (**Figure 3**). Two HFD models from independent studies (GSE43106 and GSE145665) showed noticeable differences in the DEG patterns, which may be attributed to the difference in dietary intervention duration (3 weeks vs. 24 weeks) or slightly different diet compositions (**Table 2**). However, two CDAHFD model studies (GSE120977 and GSE137449) showed relatively similar DEG patterns and were clustered together on the heatmap, indicating that upon comparable dietary intervention methods, correlative gene dysregulation patterns can be observed.

### Comparative analysis of hepatic gene signatures of human and mouse NASH

**Figure 4:**
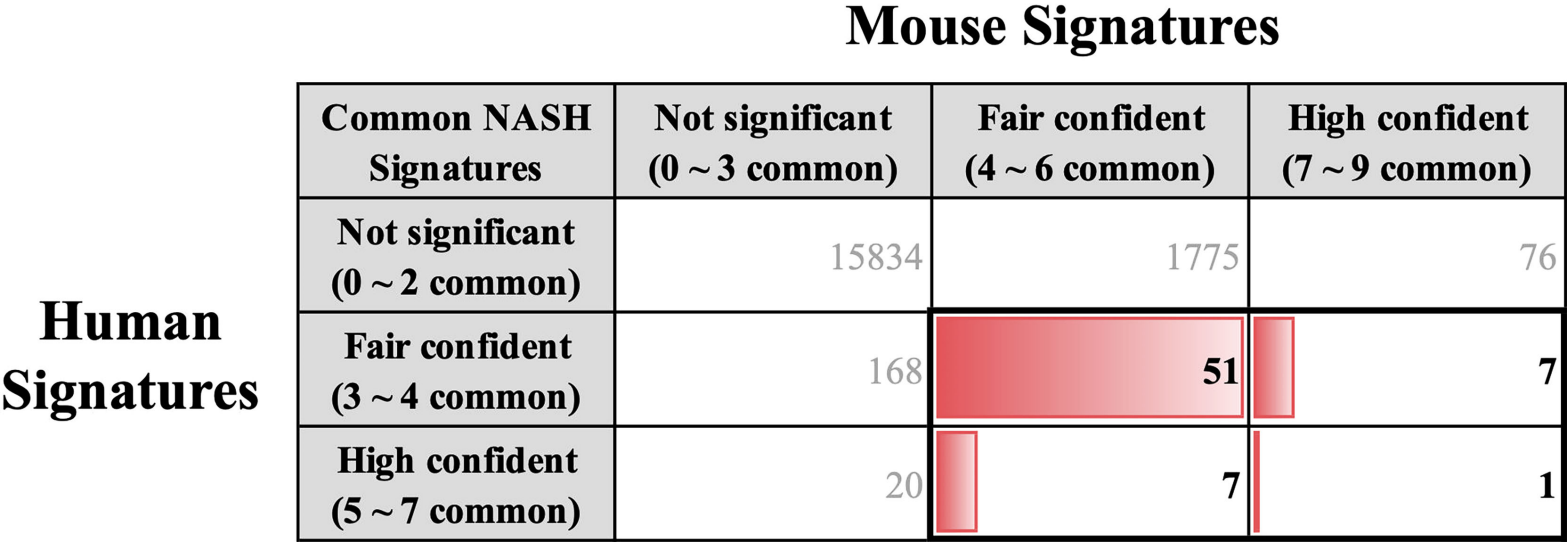
Key common hepatic signatures of human and mouse NASH. NASH signatures of humans and mice were coalesced using the BioMart orthologous gene annotation. Amongst the 254 human and 1,917 mouse NASH signatures, 66 genes (boxed with bold lines) were concordantly dysregulated between the two species.

We coalesced the NASH signatures of humans and mice using BioMart orthologous gene annotation. Combining the NASH signatures of humans and mice yielded a pattern that is similar to that observed by Teufel et al. (32). Profound differences were observed between the two signatures. Amongst the 254 human and 1,917 mouse NASH signatures, 66 genes were concordantly dysregulated between the two species (**Figure 4**). While comparing the NASH signatures, we prioritized genes that were significantly dysregulated in humans to derive clinically relevant results. Among the 28 “High confident” human NASH signatures, 8 genes were shown to be co-dysregulated in mice, whereas 20 genes did not match or showed no statistical significance in the mouse signature. In detail, CYP2C19 was excluded from the combined signature because humans and mice have different isoform variations. In mice, 10 genes (CDH23, CMYA5, EFHD1, ENO3, GPR88, PI6K3, P4HA1, PDE11A, STMN2, and VIL1) showed no evidence of dysregulation, and 7 genes (ABCB11, ACSL4, APOF, FAT1, LEPR, SOCS2, and TMEM154) showed some evidence of dysregulation, but were not statistically significant. Interestingly, ME1 and TSPAN13 showed distinctively opposite dysregulation patterns in the two species. (**Supplementary Figure 4**).

### Key common NASH signatures and protein level validation

**Figure 5:**
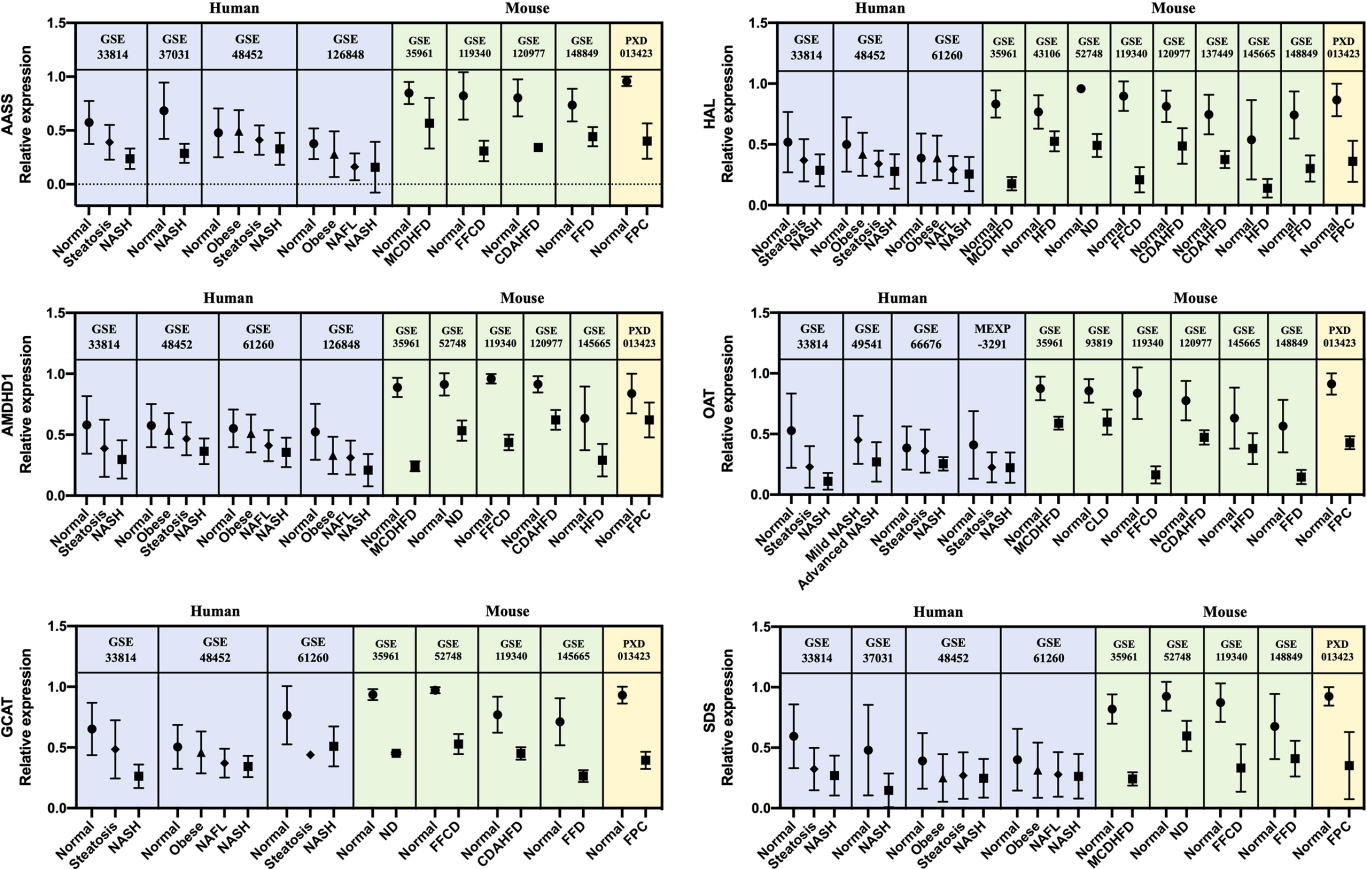
Relative gene and protein expression values of down-regulated amino acid metabolism enzymes across human and mouse datasets. Blue boxes indicate data from human transcriptome studies, green boxes indicate data from mouse transcriptome studies, and yellow boxes indicate data from mouse proteome studies. Graphs show the mean values with SD. For every dataset, *p*-value< 0.05 for normal versus NASH group. AASS, alpha-aminoadipic semialdehyde synthase; AMDHD1, amidohydrolase domain containing 1; GCAT, glycine C-acetyltransferase; HAL, histidine ammonia-lyase; OAT, ornithine aminotransferase; SDS, serine dehydratase.

A list of 66 genes co-dysregulated in human and mouse NASH is provided in the **Supplementary Data**. Notably, the gene corresponding to glycine-N-methyltransferase (GNMT) was classified as a “High confident” gene in both the human and mouse signatures. Subsequently, to validate the 66 NASH signatures at the protein expression level, we performed a meta-analysis of proteome studies of human and mouse NASH. A systematic dataset search revealed one dataset, PXD013423, which analyzed whole-liver proteome expressions from a mouse fed with fructose palmitate cholesterol (FPC) diet (31). Using the SEQUEST algorithm for MS2 level peptide spectral matching, we identified 4,679 proteins with FDR ≤ 1%. A total of 617 proteins (232 up-regulated and 385 down-regulated) was selected as the NASH DEPs (**Supplementary Figure 5**). Finally, when 66 genes were validated using the DEPs, 14 genes were confirmed to be dysregulated at the protein level (**Table 3**). At the biological process level, these 14 validated NASH signatures were strongly associated with amino acid metabolism (**Table 4**). Particularly, six enzymes involved in amino acid metabolism were significantly down-regulated in both human and mouse NASH (**Figure 5**). This dysregulation was more prominent in the mouse datasets, and NAFLD progression-dependent down-regulation was observed in human datasets.

**Table 3:** List of 14 common NASH signatures validated at the protein expression level.

Gene symbol	Gene title	Dysregulation	Human frequency score (# out of 10)	Mouse frequency score (# out of 10)	Mouse protein fold change
**AASS**	Alpha-aminoadipic semialdehyde synthase	Down	4	4	0.41
**AMDHD1**	Amidohydrolase Domain Containing 1	Down	4	5	0.56
**ANXA2**	Annexin A2	Up	3	8	3.55
**CYP1A2**	Cytochrome P450 Family 1 Subfamily A Member 2	Down	4	5	0.39
**FABP4**	Fatty Acid Binding Protein 4	Up	4	7	1.83
**GCAT**	Glycine C-Acetyltransferase	Down	3	4	0.35
**GNMT**	Glycine N-Methyltransferase	Down	6	7	0.12
**GSN**	Gelsolin	Up	3	6	1.76
**HAL**	Histidine Ammonia-Lyase	Down	3	8	0.24
**KRT19**	Keratin 19	Up	3	5	1.82
**LGALS3**	Galectin 3	Up	3	7	7.17
**LUM**	Lumican	Up	4	6	2.39
**OAT**	Ornithine Aminotransferase	Down	4	6	0.36
**SDS**	Serine Dehydratase	Down	4	4	0.34

#, number.

**Table 4:** GO BP enrichment analysis on 14 validated NASH signatures.

GO BP ID	Enriched GO BP terms in DEGs	*p*-value	# of DEGs in GO BP	# of genes in reference	DEGs
**GO:0019557**	histidine catabolic process to glutamate and formate	6.42E-06	2	4	AMDHD1, HAL
**GO:0006567**	threonine catabolic process	8.98E-06	2	5	GCAT, SDS
**GO:1901607**	alpha-amino acid biosynthetic process	1.22E-05	3	64	AASS, OAT, SDS

GO BP, gene ontology biological process; NASH, nonalcoholic steatohepatitis; DEGs, differentially expressed genes; #, number.

## Discussion

### Principal findings and corresponding interpretations

In this study, we performed a systematic meta-analysis of transcriptome studies of liver tissues from NASH patients and mouse models. As a result, we constructed 254 human and 1,917 mouse NASH gene signatures. According to the GO BP enrichment analysis, the up-regulated genes of the 254 human NASH signatures were associated with inflammation, steatosis, apoptosis, and ECM organization, which are the most significant hepatic perturbations in the pathogenesis of NASH. Down-regulated NASH signatures were associated with cellular response to metal ions, lipid metabolism, and amino acid metabolism. Interestingly, cellular responses to copper, cadmium, and zinc ions were found to be the most significantly enriched biological processes among the down-regulated gene signatures. This result was due to the consistent down-regulation of metallothionein genes– MT1E, MT1F, MT1M, MT1X, and MT2A. Metallothionein is a family of cysteine-rich, low molecular weight proteins with metal-binding capacity, thus protecting the cells from metal toxicity and oxidative stress (33). Its expression is believed to be dependent on the cellular environment of stress and mineral availability. Accordingly, accumulated evidence suggests a zinc and copper deficiency in the serum and/or hepatic tissue of NAFLD patients (34, 35). Mechanistic studies using rats also showed the causative role of zinc and copper deficiency in the pathogenesis of NAFLD (35, 36). However, despite these reports, metallothionein gene (mouse isoforms Mt1 and Mt2) dysregulation was not statistically significant in our mouse NASH signature. This may be explained by the fact that almost all experimental NASH models supply the same amounts of micronutrients (e.g., minerals) to both control and NASH-inducing groups. Although the intake of individual dietary minerals varies in clinical circumstances, mineral intake in NASH mouse models is tightly controlled. To confirm the effect of mineral deficiency and metallothionein dysregulation in the pathogenesis of NASH, dietary models with varying amounts of minerals should be used (36). Although mineral deficiencies may not be the primary cause of NAFLD, our results and those of others strongly suggest that these deficiencies are involved in the disease pathogenesis.

Mouse models are integral to the studies of NASH pathogenesis, and many different dietary methods have been developed. However, no single model has been established to represent the full spectrum of human NASH, and each model reflects different aspects of the disease. Since the transcriptome profile is believed to represent the overall biological status of cells or tissues, we hypothesized that a mouse model exhibiting the most similar gene expression profile to the 254 human NASH signatures would best represent the hepatic conditions of human NASH. Upon comparison, models with FFCD and FFD showed the strongest resemblance to the human NASH signature, each reflecting 90 of the 254 genes. Im et al. recently carried out a systematic literature review of 3,920 NAFLD mouse models and concluded that dietary models with high fat and high fructose most closely resembled the metabolic and histological features of human NAFLD (37). This is in line with our results, considering that FFCD and FFD both have large amounts of fat and fructose/sucrose as the main diet composition. These two models were followed by the models, CDAHFD-12 week (85 genes), CDAHFD-8 week (73 genes), and MCDHFD (73 genes), in terms of human NASH resemblance. Choline-deficient diet models showed the highest mean liver histology scores in a systematic review study, but had relatively poor metabolic features compared to high-fat high-fructose diet models (37). Taken together, these results indicate that our transcriptome signature is in good correlation with the metabolic and histological features of NASH. On the contrary, high-fat diets with no added fructose exhibited the least gene expression features of human NASH in our study, which also highlights the importance of fructose in the pathogenesis of NASH.

Transcriptomics is a powerful tool that can measure genome-scale mRNA expression levels with high accuracy, and thus infer protein abundances within a biological system. However, microarray or RNA-seq experimental results still require follow-up validation. Hence, we performed a meta-analysis of proteomic studies of human and mouse NASH, and derived liver tissue DEPs from the FPC NASH mouse model. When 66 common NASH signatures were validated using the DEPs, 14 genes were confirmed as dysregulated at the protein level. Among the 14 genes, those such as FABP4, GNMT, and LUM are well-known to be associated with NASH (38–40), and galectin-3 encoded by LGALS3 is currently being tested as a therapeutic target in a phase 2b/3 clinical trial (41). Inclusion of these prominent drug targets motivated us to investigate other relatively less-studied genes.

Notably, among the 14 genes, down-regulation of AASS, AMDHD1, GCAT, HAL, OAT, and SDS was associated with amino acid metabolism in the GO BP enrichment analysis. In particular, histidine and threonine catabolic processes and α-amino acid anabolic processes were prominently enriched. Several previous studies have corroborated our findings. Lake et al. performed metabolome analyses on liver tissues from NAFLD patients and reported an increased level of lysine in NASH patients (18). Alpha-aminoadipic semialdehyde synthase encoded by AASS is involved in the first two steps of lysine degradation *via* the saccharopine pathway within the mitochondria, and down-regulation of AASS may lead to elevated levels of lysine in the liver. Eriksen et al. assessed the expression of urea cycle-related genes in NAFLD patients and reported the down-regulation of hepatic genes governing ureagenesis as well as impaired amino acid metabolism (42). Down-regulation of hepatic α-amino acid metabolism was manifested by higher blood concentration of α-amino nitrogen levels in NAFLD patients (42). In our study, down-regulation of AMDHD1 and HAL, which are involved in the catabolic pathway of histidine to glutamate conversion, and down-regulation of AASS, which also produces glutamate along the saccharopine pathway, may be presumed to result in glutamate level alteration in NASH. Accordingly, a metabonomic study by García-Cañaveras et al. reported decreased levels of glutamate in human NAFLD liver tissues (43). Glutamate plays a critical role in hepatic amino acid metabolism, acting as a key intermediate between the urea cycle and citric acid cycle, and it is a major substrate for glutathione (GSH) synthesis (44). Decreased level of glutamate in the liver can compromise the replenishment of GSH and make the liver more susceptible to pathological environment. In short, our results of gene dysregulation analysis reflect the metabolic status of the NAFLD liver. Interestingly, the down-regulation of amino acid metabolism enzymes was associated with NAFLD progression in human datasets. To the best of our knowledge, this is the first study to report six significant enzymes responsible for the dysregulation of amino acid metabolism in the pathogenesis of NASH.

### Effect of the number of included datasets in meta-analysis

In this study, we used 10 human transcriptome datasets to establish NASH signatures. To determine whether the number of included datasets affects the overall NASH signatures, we randomly selected five human datasets (GSE37031, GSE49541, GSE63067, GSE126848, MEXP-3291) and repeated the meta-analysis. This time, the human NASH signatures consisted of 328 DEGs (DEGs with frequency ≥ 2). When 328 NASH signatures were compared with the previous 254 NASH signatures, 171 DEGs were found to be common (**Supplementary Figure 6**). Interestingly, there were some discrepancies between the two signatures. To explain this discrepancy, we provide examples of two DEGs, ABCC4 and AMDHD1, which were unique in each NASH signature. When we analyzed the transcriptional profile for ABCC4 and AMDHD1 across the 10 human datasets, ABCC4 was upregulated in the GSE126848 and MEXP-3291 datasets, whereas AMDHD1 was downregulated in the GSE33814, GSE48452, GSE61260, and GSE126848 datasets. Therefore, ABCC4 could be selected as NASH signatures when only five datasets were used (DEGs with frequency ≥ 2), but lacked statistical significance when all 10 datasets were used (DEGs with frequency ≥ 3). In contrast, AMDHD1 was selected as a NASH signature when 10 datasets were used, but the frequency score decreased to 1 when five datasets were randomly selected (GSE33814, GSE48452, and GSE61260 were not selected in this case). This result strongly supports the importance of using a comprehensive dataset when performing a meta-analysis. In our study, at least six datasets were required to obtain a threshold DEG frequency score of 3, but this result may vary according to the characteristics of each included dataset and the number of DEGs selected.

### Comparison with other studies

We identified two studies that performed a large-scale systematic meta-analysis of human NASH transcription profiles and which they derived NASH gene signatures. The study by Ryaboshapkina and Hammar performed meta-analysis of seven microarray datasets and extracted 218 gene signature that are affected during the NAFLD progression (45). Jia and Zhai integrated six microarray datasets and established 96 significant DEGs between healthy people and NAFLD patients, using the robust rank aggregation method (46). In our study, we performed a comprehensive meta-analysis of 10 microarray and RNA sequencing datasets, and derived 254 statistically confident human NASH signatures. While comparing our NASH signature with those of the other two studies, we found five genes–FABP4, GNMT, IL32, TP53I3, and VIL1–to be common (**Supplementary Figure 7**). However, the overall signatures of each study were relatively different, and 181 genes (37.1%) were unique to our study. The differences in inclusion criteria during the dataset selection process, number of included datasets, types of included assay platforms, and different statistical approaches may all contribute to the diversity of individual gene signatures.

### Limitations

Our study had several inherent limitations. First, while analyzing human transcriptome studies, disease progression variables were not considered. Although all study participants were diagnosed histologically, inter-observer variation exists in NASH diagnosis, and each study used different patient classifications. To minimize the effect of disparity on disease assessment between study cohorts and to systematically compare gene expression profiles from multifarious datasets, DEGs were selected by comparing normal subjects and well-defined NASH patient groups. Second, participant baseline characteristics and clinical biochemical data analyses were not possible due to limitations in data acquisition. Third, while combining human and mouse NASH signatures, genes with no reported orthologs and isoforms of genes that are unique to either species were excluded during the process. Finally, protein-level validation of NASH signatures was only performed using the mouse proteome dataset. Datasets of the whole liver proteome from NASH patients were not available, which strongly suggests a need for further studies.

## Conclusions

We performed a meta-analysis of NASH transcriptome datasets and established the most comprehensive 254 human and 1,917 mouse NASH signatures to date. Based on comparison of different dietary models with the human NASH signature, our results add to the existing body of literature suggesting that dietary models with high fat and high fructose most closely resemble the genetic, metabolic, and histological features of human NAFLD. Cross-species analysis revealed 66 genes to be concordantly dysregulated between human and mouse NASH. Among these, 14 genes were further validated to be dysregulated at the protein level. The resulting 14 genes included some well-established NASH-associated genes and a promising NASH drug target. Functional enrichment analysis demonstrated that dysregulation of amino acid metabolism was the most significant hepatic perturbation in both human and mouse NASH. Moreover, down-regulation of six amino acid metabolism enzymes, AASS, AMDHD1, GCAT, HAL, OAT, and SDS, was associated with NAFLD progression in humans. Further studies are needed to unravel the link between dysregulation of hepatic amino acid metabolism and NAFLD pathogenesis.

## Data availability statement

All datasets analyzed in this meta-analysis are publicly available from respective data repositories. The final meta-analysis result of human and mouse transcriptome–proteome profile data is provided in the Supplementary Data.

## Author contributions

All authors listed have made substantial, direct, and intellectual contribution to the work and approved it for publication. All authors of this paper have read and approved the final version.

## Funding

This work was financially supported by the Korea Institute of Science and Technology under grant (2Z06661) and (2Z06666).

## Acknowledgments

We appreciate the authors of the transcriptomic and proteomic studies for making their data publicly available and all the patients who contributed to the research. We would like to offer special thanks to Yura Song for providing technical advice on transcriptome analyses and Moonyoung Lee for providing advice on statistical analyses.

## Conflict of interest

The authors declare that the research was conducted in the absence of any commercial or financial relationships that could be construed as a potential conflict of interest.

## Publisher’s note

All claims expressed in this article are solely those of the authors and do not necessarily represent those of their affiliated organizations, or those of the publisher, the editors and the reviewers. Any product that may be evaluated in this article, or claim that may be made by its manufacturer, is not guaranteed or endorsed by the publisher.
