# Supplementary material for: Key hepatic signatures of human and mouse nonalcoholic steatohepatitis: A transcriptome–proteome data meta-analysis
Source: Front Endocrinol (Lausanne). 2022 Oct 4;13:934847. doi: 10.3389/fendo.2022.934847 (PMC9576953; doi:10.3389/fendo.2022.934847)
Supplement: Supplementary file 2 [file DataSheet_2.docx]

Supplementary Figures


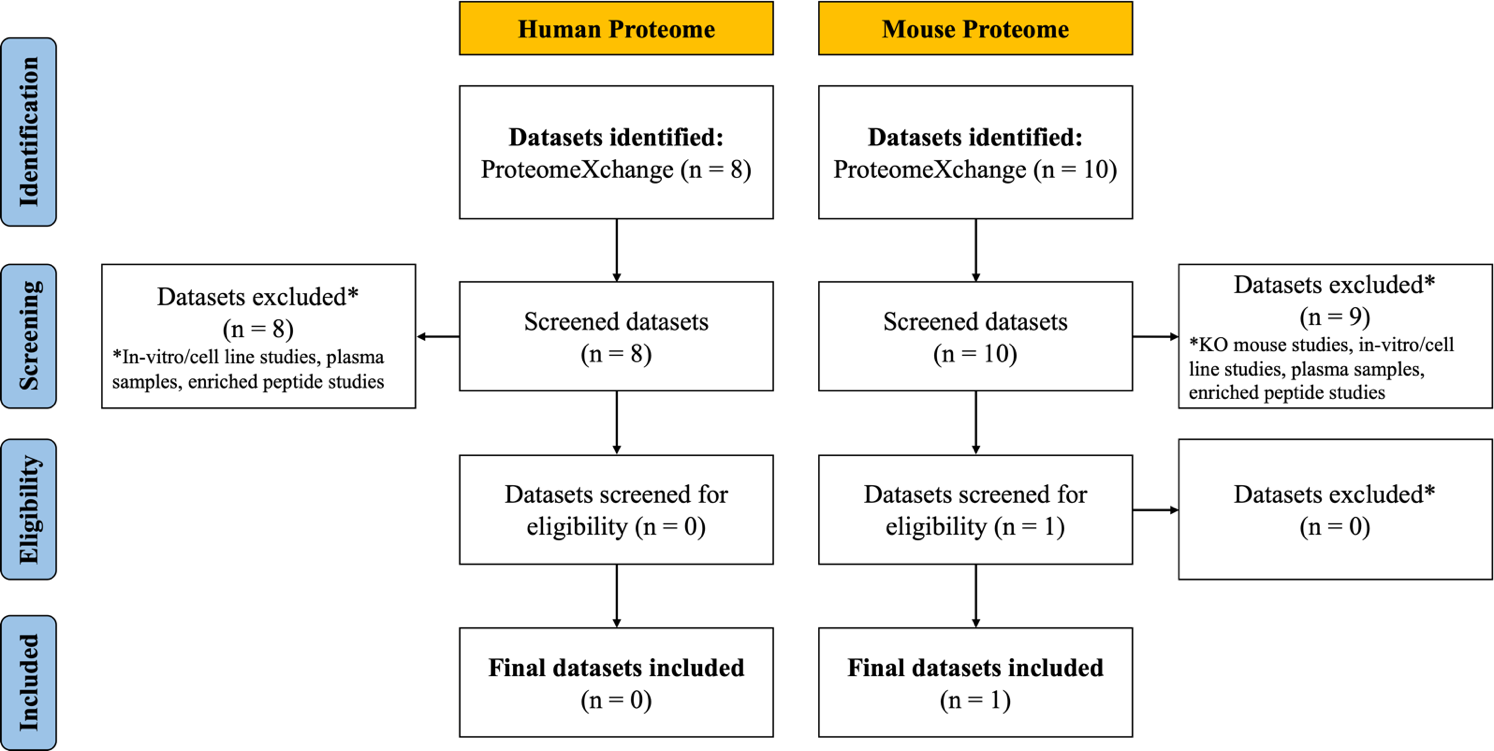


**Supplementary Figure 1.** Flow diagram on the selection process of human and mouse NASH proteome datasets.


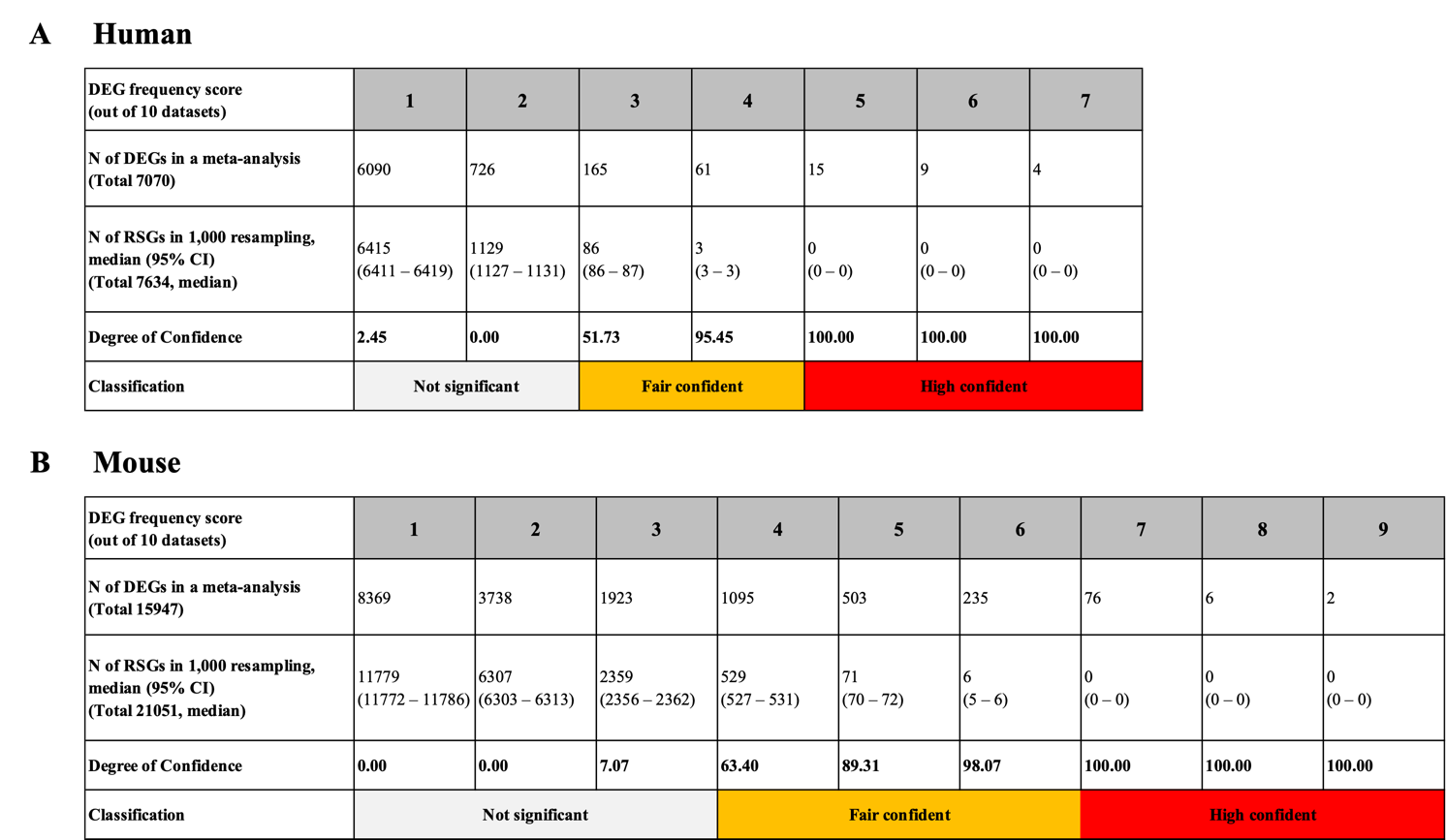


**Supplementary Figure 2.** Degree of confidence of DEGs per DEG frequency score in multiple transcriptome dataset meta-analyses for (A) human and (B) mouse studies. Values under 0 were denoted as 0. DEGs with degree of confidence less than 50 were classified as “Not significant,” confidence between 100 and 50 were classified “Fair confident,” and confidence of 100 were classified “High confident” genes. Abbreviations: N, number; DEGs, differentially expressed genes; RSGs, randomly selected genes; CI, confidence interval.


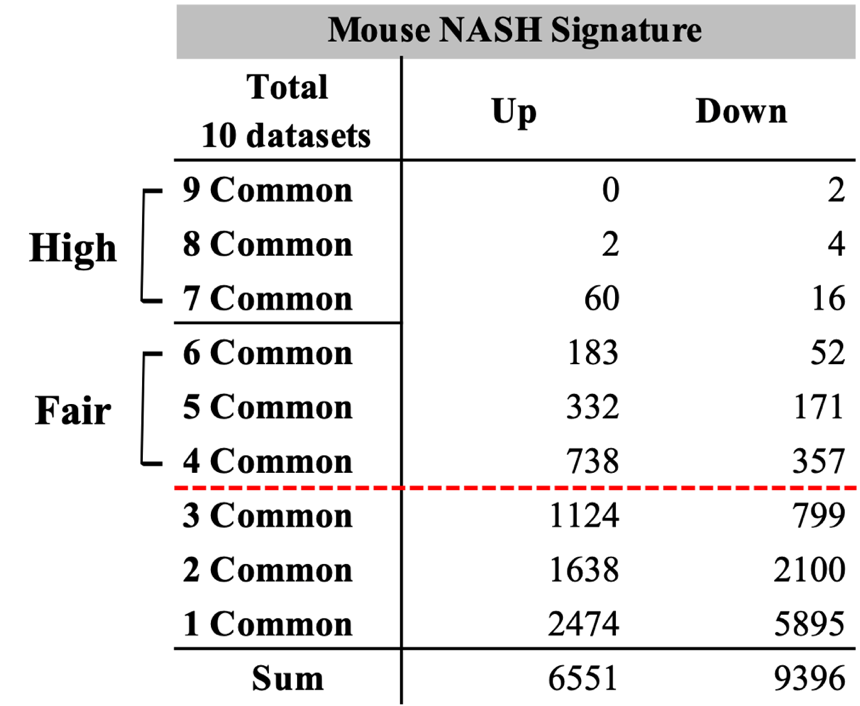


**Supplementary Figure 3.** Establishment of mouse NASH gene signatures. Individually selected DEGs from 10 mouse NASH datasets were merged based on the gene symbols and frequency score of each DEG was calculated. Total 1,917 genes (1,315 up-regulated and 602 down-regulated) were consistently dysregulated in at least 4 datasets. DEGs with frequency score of 4 to 6 were classified as “Fair confident” (chance finding probability ≤ 50%) and frequency score of 7 to 9 were classified as “High confident” (chance finding probability ≤ 1%) genes.


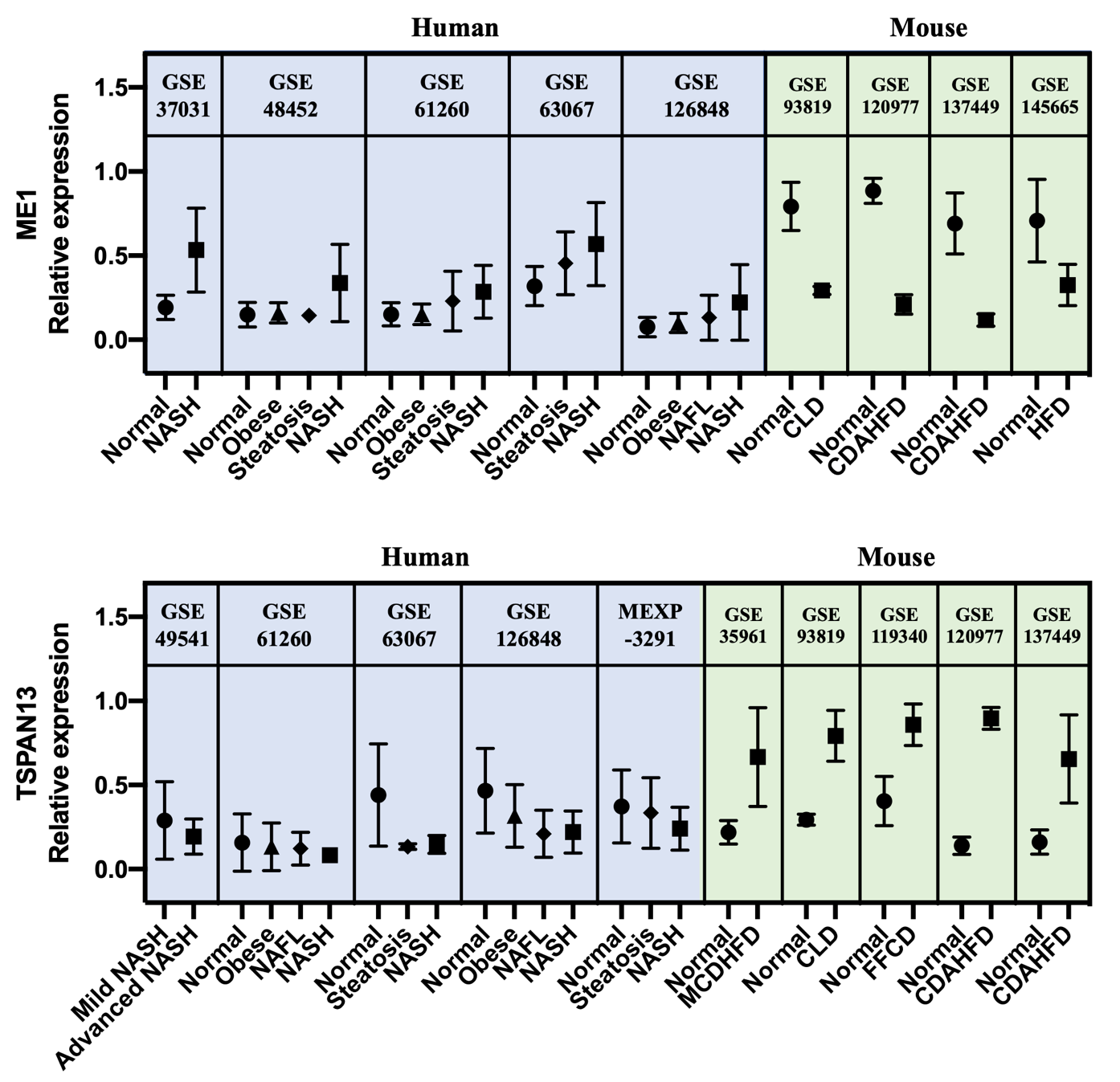


**Supplementary Figure 4.** Relative gene expression values of ME1 and TSPAN13 across human and mouse datasets. Blue boxes indicate data of human transcriptome studies, and green boxes indicate data of mouse transcriptome studies. Graphs show mean values with SD. For every dataset, *P*-value < 0.05 for normal versus NASH group. Abbreviations: ME1, NADP-dependent malic enzyme; TSPAN13, tetraspanin 13.

**
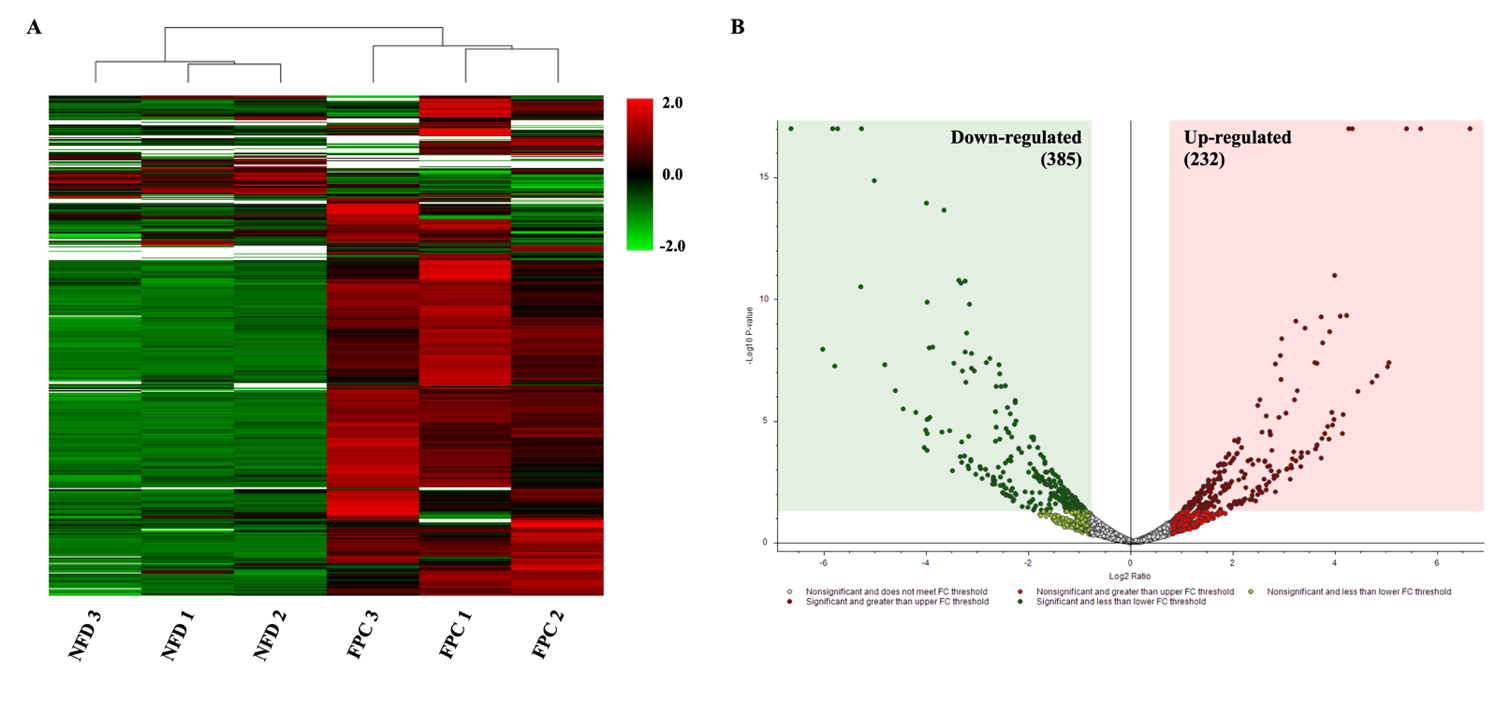
**

**Supplementary Figure 5.** NASH mouse model liver proteome study analysis. The RAW data of FPC-induced NASH model proteome study PXD013423 was downloaded from ProteomeXchange. (A) Proteome expression profiles of NFD and FPC groups were analyzed using the heatmap and (B) differentially expressed proteins were selected using the SEQUEST search parameters. NFD, normal feed diet; FPC, fructose palmitate cholesterol diet.

**Supplementary Figure 6.** Comparison of NASH signatures that were derived from using 5 datasets and 10 datasets.


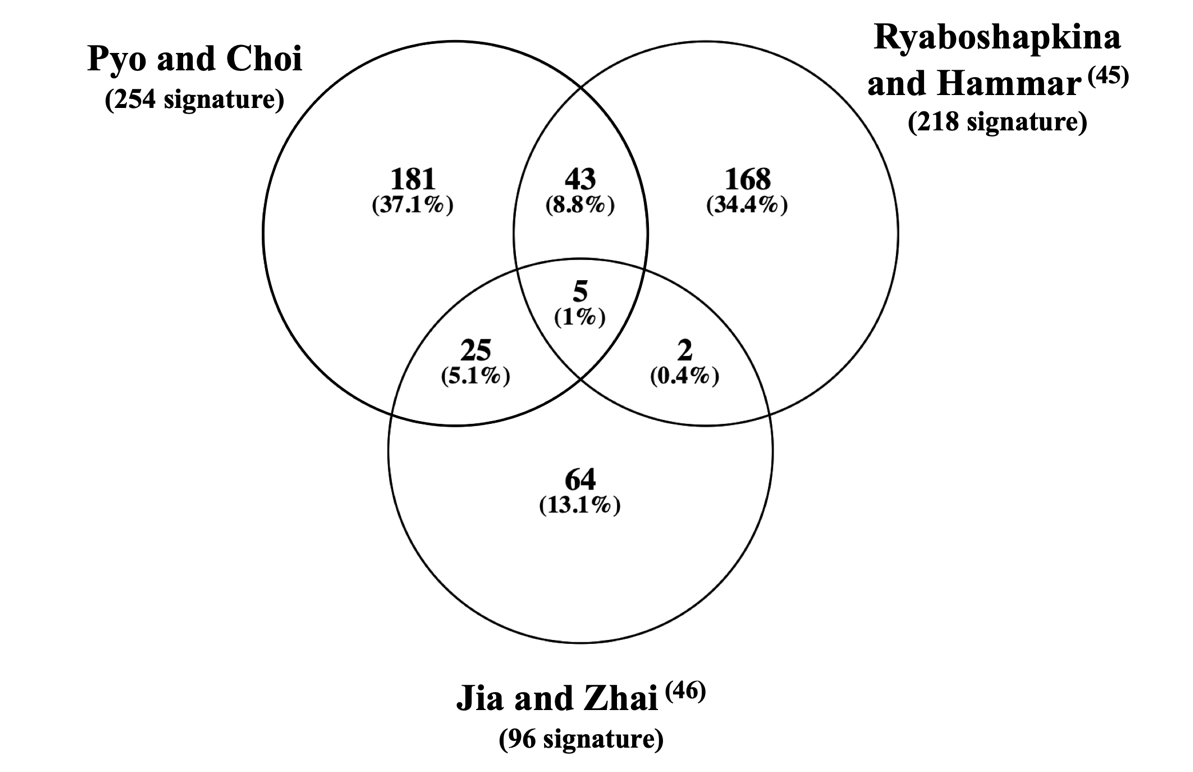


**Supplementary Figure 7.** Comparison with other human NASH transcriptome meta-analysis studies.
